# Improvement of the Meat Quality of Holstein Bulls Fed a Diet Enriched with Oregano Oil

**DOI:** 10.3390/ani14233408

**Published:** 2024-11-26

**Authors:** Anestis Tsitsos, Stella Dokou, Tryfon Chatzimanou, Ilias Giannenas, Vangelis Economou, Georgios Arsenos

**Affiliations:** 1 Laboratory of Animal Food Products Hygiene—Veterinary Public Health, School of Veterinary Medicine, Aristotle University of Thessaloniki, 541 24 Thessaloniki, Greece; tsitanes@vet.auth.gr; 2 Laboratory of Animal Nutrition, School of Veterinary Medicine, Aristotle University of Thessaloniki, 541 24 Thessaloniki, Greece; dokoustella@vet.auth.gr (S.D.); igiannenas@vet.auth.gr (I.G.); 3 Laboratory of Anatomy, Histology and Embryology, School of Veterinary Medicine, Aristotle University of Thessaloniki, 541 24 Thessaloniki, Greece; tryfoncc@vet.auth.gr; 4 Laboratory of Animal Husbandry, School of Veterinary Medicine, Aristotle University of Thessaloniki, 541 24 Thessaloniki, Greece; arsenosg@vet.auth.gr

**Keywords:** cattle, beef, microbial count, chemical composition, organoleptic evaluation, color values, lipid oxidation, fatty acids, amino acid profile

## Abstract

Beef is a dominant meat commodity produced mainly from beef cattle breeds. Dairy cattle breeds are reared for milk production, but their calves could be raised to produce high quality meat subject to designated feeding. The objective here was to investigate the role of oregano oil-enriched diets on the quality characteristics of meat produced by Holstein bulls. Fifty Holstein bulls, aged 12 ± 2 months and with an average weight of 365 ± 60 kg, were used. They were equally allocated in two experimental groups (n = 25). One group (group CON) was offered a basal diet and the other (group OREG) the same diet enriched with 50 mg/kg DM of oregano oil, for a period of 90 days. Afterwards, meat samples were subjected to microbiological (1 h after slaughter), physicochemical (1st and 15th day after slaughter), and sensory analyses (15th day after slaughter). The beef of group OREG is characterized with improved color parameters and a lower malondialdehyde (MDA) production rate and content (68.52 ng/g versus 105.91 ng/g, *p* = 0.01). The sensory evaluation of beef revealed higher scores for group OREG in flavor (3.88 versus 3.17, *p* = 0.0007) and color (3.87 versus 3.21, *p* = 0.001), as well as higher nutritional value, which was associated with significantly higher levels of alanine, arginine, aspartic acid, histidine, isoleucine, leucine, lysine, valine, and total amino acids (*p* < 0.05). Thus, the results suggest that the quality of the meat of Holstein bulls can be improved when they are fed a diet enriched with oregano oil.

## Simple Summary

The objective of this study was to evaluate the potential benefits of an oregano oil-enriched diet on improving the meat quality of Holstein bulls. Two groups of twenty-five Holstein bulls each, with an average of 12 months of age, were fed either a basal diet or the same diet enriched with 50 mg/kg DM of oregano oil for a period of 90 days. After slaughter, meat samples were analyzed for microbiological, physicochemical, and sensory properties. The meat from bulls fed the oregano oil-enriched diet exhibited improved sensory attributes (notably flavor and color, with the latter confirmed by instrumental analysis), a lower content and rate of malondialdehyde production, and a higher nutritional value, particularly in amino acid content. These findings suggest that including oregano oil in the diet of Holstein bulls can improve their meat quality.

## 1. Introduction

Beef is one of the most widely consumed types of meat globally [1] with bovine meat production reaching 76.6 million tonnes in 2023 [2]. It plays an important role in human nutrition, being rich in essential nutrients. It is rich in high-quality proteins, essential amino acids, vitamin B complex, essential minerals, and contains polyunsaturated fatty acids (PUFAs), such as linoleic acid and α-linolenic acid [3,4]. While its nutritional value is well established, its role in human nutrition is recently debated in terms of safety and quality parameters. The safety aspect, a fundamental requirement in food consumption, aligns with the subjective nature of quality, which is influenced by consumer preferences [1,5]. Consumer preferences are shaped by both intrinsic (i.e., psychological, social, and cultural influences) and extrinsic factors (i.e., marketing and packaging) [1,6]. The objective assessment of meat quality using analytical methods involves certain physicochemical parameters, such as pH, tenderness, and chemical composition, that may not be readily perceived by consumers [6]. In the context of beef, there is a considerable variation in demand and quality due to diverse production systems and rearing methods influenced by climate and topographical differences throughout the year [7,8,9]. Consequently, the characterization of meat quality often relies on empirical characteristics that vary among consumers.

Natural feed additives, such as probiotics, enzymes, and plant derived products, are introduced as an environmentally friendly, and highly accepted by consumers, strategy to improve beef productivity and quality [10]. Among them, essential oils are commonly incorporated in animal diets and are often used by food industries, in order to improve the quality characteristics of meat and to enhance its shelf life. A recent meta-analysis evaluating the effects of essential oils on small ruminant meat quality showed beneficial effects, including reduced meat malondialdehyde levels and lower total viable bacterial counts [11]. Likewise, a meta-analysis on the dietary inclusion of essential oils in beef cattle showed consistent results regarding the positive impact of essential oils on meat oxidation status [12]. Both studies support the promising effects of essential oils as natural feed additives on meat quality. Oregano oil is an essential oil derived from the plant *Origanum vulgare* and is considered food graded (GRAS) by the Food and Drug Administration (FDA) [13], whereas the European Food Safety Authority (EFSA) Panel on Food Additives and Nutrient Sources has suggested a dietary intake [14]. Oregano essential oil is known for its rich composition of bioactive molecules, including carvacrol, thymol p-cymene, and γ-terpinene that contribute to its therapeutic properties [15]. It exhibits considerable biological properties such as antimicrobial, anticoccidial, antifungal, antiviral, and antioxidant. Moreover, it also plays a role in digestion aid, including stimulation of endogenous enzyme activity, nitrogen absorption, inhibition of odor, ammonia control, and stimulation of the immune response [16,17]. Its antimicrobial and antioxidant properties are attributed mainly to its constituents carvacrol and thymol [13]. Oregano oil can be used directly to food products or incorporated into animal diet used along with stabilizing methods, such as incorporation into emulsions, liposomes, and capsules, to overcome the hydrophobic nature of oregano oil and to enhance its bioactive effects [18,19]. In animal nutrition, it has been used as a performance enhancer to manipulate microbial metabolism and to improve feed efficiency, animal productivity, and food quality [20].

Dairy cattle are reared for milk production and the meat produced by their offsprings is considered of lower quality, especially when compared to beef cattle breeds, such as Angus and Limousin [21,22]. However, the notion is that their offsprings can produce high quality meat subject to appropriate feeding management [23,24]. There are numerous studies regarding the impact of oregano oil-enriched diets on the performance parameters of monogastric animals and the quality characteristics of produced meat, such as poultry and pork [16,25]. Thus, this study’s hypothesis is that oregano oil could enhance beef quality characteristics, as it has been shown to improve the quality traits of meat produced by monogastric animals. However, only a few of them explored the use of oregano oil in cattle nutrition, especially dairy cattle breeds, to improve the quality parameters of beef. Hence, the objective here was to investigate the role of oregano oil-enriched diets on the quality characteristics (microbiological, physicochemical, and organoleptic traits) of meat produced by Holstein cattle.

## 2. Materials and Methods

### 2.1. Experimental Design

Fifty Holstein bulls, initially weighing 365 ± 60 kg and aged 12 ± 2 months, were utilized. The experiment was conducted on a commercial farm in Northern Greece. The bulls were allocated into two groups following a randomized blocked design. Each group was accommodated in five feedlot pens, allowing 7 m^2^ per bull. Following a 14-day acclimatization period, bulls were offered a diet designed to meet maintenance and growth requirements. Table 1 shows the composition of the basal diet used for the control group (CON). In the test group (OREG), the basal diet was supplemented with 50 mg/kg of oregano oil (containing 97.95% carvacrol and 0.75% thymol). The oregano essential oil was produced by steam distillation of cultivated oregano (*Origanum vulgare hirtum*) from Northern Greece. The concentration of oregano oil in OREG was well below the limit of 88 mg/kg for fattening cattle as proposed by EFSA’s Panel on Additives and Products or Substances used in Animal Feed (FEEDAP) [26]. Two feeds were formulated and offered over a period of 90 days. Feed was provided as a total mixed ration twice daily (08:00 and 17:00), and water was available ad libitum. After the end of the experiment, the bulls were slaughtered in a nearby abattoir in Northern Greece. Several samples were collected from the carcasses for microbiological, physicochemical analysis and organoleptic evaluation.

The study was conducted according to the guidelines of the Declaration of Helsinki and approved by the Research Committee of the Aristotle University of Thessaloniki (96813/3 May 2021).

### 2.2. Microbiological Analysis

The surface of each carcass was sampled using the non-destructive swab method, approximately 1 h after slaughter. More specifically, a sterile swab soaked in 5 mL of Maximum Recovery Diluent (MRD, Oxoid Ltd., Basingstoke, UK), along with a dry sterile swab, was used to wipe the surface of the carcasses. Both swabs were then placed in a tube containing 5 mL of MRD and transported in a portable refrigerator (≤4 °C) to the Laboratory for further analysis. Within 24 h, the tubes were brought to room temperature, thoroughly vortexed, and decimal dilutions were prepared in MRD-containing tubes. The sample microbial analyses concerned Total Mesophilic Viable Counts (TMVCs), Total Psychrophilic Counts (TPVCs), and coliform counts, according to methods from the International Organization for Standardization (ISO 4833/2005 [27] and ISO 21528-2/2017 [28], with modifications), and based on Commission Regulation (EC) No. 2073/2005 [29] on microbiological criteria for foodstuffs. From each dilution, 1 mL was surface inoculated onto appropriate media. Enumeration of TMVCs and TPVCs was performed on Plate Count Agar (Biolab Diagnostics, Budapest, Hungary). For coliform counts, Violet Red Bile Glucose Agar (Biolab Diagnostics, Budapest, Hungary) was used. Incubation was conducted at 30 °C for 72 h for TMVCs, 7 °C for 7 days for TPVCs, and 37 °C for 24 h for coliform counts. After incubation under appropriate conditions, the characteristic colonies were counted, and the results were recorded.

### 2.3. Physicochemical Analysis

Meat samples were collected from cold carcasses, as previously described by Tsitsos et al. [30], to evaluate the physicochemical characteristics of beef. Briefly, approximately 200 g of meat samples were collected from the 13th rib steak and transported to the laboratory on the same day. The samples were stored in vacuum packaging under refrigeration (≤4 °C) for 15 days. The physicochemical analysis included the following: (a) measurements of pH, (b) chemical composition of meat (moisture, total fats, total proteins, collagen, salt, and ash), (c) lipid oxidation, (d) color evaluation and (e) analysis of fatty acids and the amino acid profile of meat samples. The analyses were performed on the 1st day (24 h after slaughter) and the 15th day of storage. The chemical composition, as well as fatty acids and the amino acid profiles, were only evaluated on the 15th day. The samples were comminuted before evaluation using a Waring laboratory blender.

For the pH analysis, 10 g of muscle were dispersed in 40 mL of distilled water and allowed to settle. Measurements of pH were collected using a Hanna Ph211 568 pH Meter. The chemical composition of meat was determined by weighing 100 g of meat and placing it on a plastic sample pan. It was then analyzed with a near-infrared spectrometer (NIR, Perten DA7250, Perkin Elmer Ltd., Waltham, MA, USA) calibrated for meat and meat products measurement. Methods approved by ISO and the Association of Official Agricultural Chemists (AOAC) were used for NIR calibration (Soxhlet for fat, drying cabinet for moisture, Kjeldahl for protein, hydroxyproline for collagen, muffle furnace for ash, and ICP-MS for salt), with calibration models developed applying Artificial Neural Networks (ANN) and Honigs Regression™ to ensure accuracy and reliability.

Lipid oxidation was assessed using the Thiobarbituric Acid Reactive Substances (TBARS) method to calculate malondialdehyde (MDA) content in meat samples [31]. More specifically, 1 g of muscle was transferred to a plastic test tube. Then, 8 mL of 5% trichloroacetic acid (TCA) and 5 mL of 0.8% butylated hydroxytoluene (BHT) were added. The solution was homogenized using the Ultra-Turrax T25 device (Janke and Kunkel, IKA Labortechnik) for 15 min. Afterwards, the samples were centrifuged for 3 min at 3000 rpm. After centrifugation, two liquid phases were formed, separated by a solid layer. Using an automatic pipette, 2.5 mL of material was taken from the lower phase and transferred to a new plastic test tube, where 1.5 mL of 0.8% thiobarbituric acid (TBA) was added. Subsequently, the samples were heated in a water bath at 70 °C for 30 min. After heating, the absorbance of each sample was determined at 532 nm with a spectrophotometer (Shimadzu, Kyoto, Japan). The determination of MDA was based on a standard curve (1,1,3,3-tetra-ethoxypropane), and the values are expressed as ng MDA/g sample.

The color evaluation of the meat samples was performed according to Tsitsos et al. [32]. More specifically, the assessment of meat color was conducted on freshly exposed samples immediately after unpacking. The Konica Minolta CR-410 Chroma Meter was used, with a 50 mm aperture size, illuminant C, and a 2° observer. Calibration was performed with a white tile (Y: 94.8/X: 0.3130/y: 0.3190). Each meat sample was scanned three consecutive times at different positions, perpendicular to the myofibrils and avoiding fat and connective tissue. The measured values of lightness (L*), redness (a*), and yellowness (b*) were averaged for each sample. Additionally, the chroma and hue angle values of each sample were calculated, based on the averages of a* and b*, using the following formulas, as described by AMSA [33]:

chroma = (a*^2^ + b*^2^)^1/2^

hue angle = arctangent (b*/a*)

Fatty acids were extracted using the Soxtherm Soxhlet Extraction System, according to the AOAC Method 991.36, in order to define the total amount of saturated (SFAs), monounsaturated fatty acids (MUFAs) and PUFAs in beef. More specifically, the extracted fatty acids underwent trans-esterification in a methanolic potassium hydroxide solution. The resulting fatty acid methyl esters were analyzed via GC-FID. Chromatographic analyses were conducted on a Shimadzu GC-2010 Plus High-End gas chromatography system, equipped with an FID detector and a Supelco SP2560 column (100 m × 0.25 mm × 0.20 μm). Helium of 99.999% purity served as the carrier gas at a flow rate of 2 mL/min. The injection volume was 1 μL with a split ratio of 1:50. The injector temperature and the detector temperature were set at 250 °C. The temperature program followed a sequence: the initial oven temperature was 110 °C (maintained for 7 min), followed by a gradual increase at 3 °C/min to 190 °C (held for 2 min). Subsequently, a multi-step process involved a 0.5 °C/min increase to 205 °C, a 5 °C/min increase to 230 °C (held for 5 min), and a final 5 °C/min increase to 240 °C (maintained for 5 min). The total run time was 82.67 min.

The analysis of amino acids was conducted using an LC-MS/MS system. Firstly, the meat samples were hydrolyzed in a 7 N aqueous solution at 125 °C. After hydrolysis, the samples were collected in a 0.1 N HCl aqueous solution. The electrospray ionization (ESI) method was chosen for analysis due to the zwitterionic nature of amino acids, in which they can carry both positive and negative charges depending on the pH of the aqueous solution. LC–MS analysis was performed for each amino acid with [M+H]+ as the detection ion. A flow rate of 0.3 mL/min and an XR-ODS column were employed, utilizing a gradient of water and acetonitrile as mobile phases. The ionization mode was set to ESI positive, and the scan range was configured to *m*/*z* 50–1000.

### 2.4. Organoleptic Evaluation

The consumer evaluation of meat samples was performed according to Tsitsos et al. [32] with modifications. More specifically, the 13th rib steak from each animal was transported to the Laboratory and stored in vacuum packaging under refrigeration for 15 days. The organoleptic evaluation was performed on the 15th day of storage. More specifically, each steak was cooked on a pre-heated water bath at 75 °C for 10 min, until its internal temperature reached 70 °C. It was then allowed to rest for 10 min and cut into cubes, approximately 25 × 25 × 25 mm in size and 25 g in weight. The samples were served at room temperature (20 ± 2 °C) on white paper plates, each with a three-digit code. The test panel consisted of 15 individuals: 6 males and 9 females, aged between 25 and 48 years. All examiners received training in meat organoleptic evaluation. The examiners were asked to rinse their mouth with mineral water and eat unsalted bread between samples. The samples were evaluated using Hedonic Evaluation, focusing on the purchasing decision and overall product acceptance. The parameters assessed included color, flavor, taste, texture, and Overall Acceptance on a five-point scale (1. Dislike very much, 2. Dislike, 3. Neither like nor dislike, 4. Like, 5. Like very much).

### 2.5. Statistical Analysis

The statistical analysis of the data involved the use of parametric and non-parametric statistical methods. Regarding the parametric methods, the Independent Samples *t*-test was used to compare the examined variables between Group OREG and Group CON. Additionally, the Paired Samples *t*-test was used to assess the change in the examined parameters over time. The normality of the data was checked using the Shapiro–Wilk test, while the homogeneity of variances was assessed using the Levene’s test. The Mann–Whitney test was used to compare the results of the sensory evaluation and fatty acids between the meat samples of Group OREG and Group CON. Furthermore, measures of central tendency and dispersion were calculated for each variable to reveal the characteristics of the data. The significance level was set at 5% (*p* ≤ 0.05). The statistical analysis was conducted using the statistical software program IBM SPSS Statistics (v.29.0., IBM Corporation, Armonk, NY, USA).

## 3. Results

### 3.1. Microbiological Analysis

**Figure 1:**
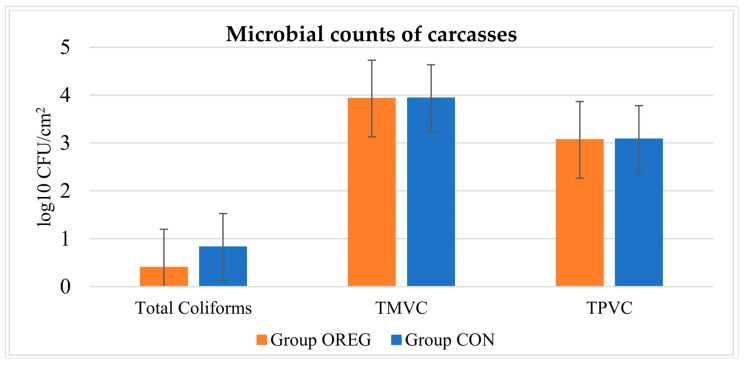
Microbial analyses (Total Mesophilic Viable Count, TMVC; Total Viable Psychrophilic Count, TMPC; Total Coliforms) of the carcasses of the test group (OREG) and the control group (CON).

The microbiological results are depicted in Figure 1. TMVCs were 3.92 (±0.87) log_10_ CFU/cm^2^ and 3.93 (±0.69) log_10_ CFU/cm^2^, TPVCs were 3.06 (±0.90) log_10_ CFU/cm^2^ and 3.07 (±0.75) log_10_ CFU/cm^2^, and total coliform counts were 0.40 (±0.43) log_10_ CFU/cm^2^ and 0.82 (±0.92) log_10_ CFU/cm^2^, in Group OREG and Group CON, respectively. No correlation was observed between TMVCs, TPVCs, and the total coliform counts of the carcasses (*p* > 0.05). Moreover, no significant differences were observed between the microbial populations of carcasses from Group OREG and Group CON (*p* > 0.05).

### 3.2. Physicochemical Analysis

The results of the chemical composition and pH of beef are presented in Table 2 and Table 3. The beef in Group OREG and Group CON had an average moisture content of 73.87% and 73.77%, total fat content of 2.65% and 2.57%, and total protein content of 22.14% and 22.5%, respectively. Moreover, on the 1st and 15th day of storage, the mean pH value of meat from Group OREG was 5.61 and 5.62, whereas the mean pH value of meat from Group CON was 5.56 and 5.58, correspondingly.

Regarding lipid oxidation, meat from Group OREG had statistically lower amounts of MDA, compared to meat from Group CON (*p* = 0.01 < 0.05) on the 15th day of storage. More specifically, the MDA content of beef was 33.31 ng/g, 68.52 ng/g (Group OREG), and 47.92 ng/g, 105.91 ng/g (Group CON), on the 1st and 15th day of storage, respectively. The concentrations of MDA in beef from both groups showed a significant increase during the storage period. The results of lipid oxidation of beef are presented in Table 3.

Concerning color evaluation, beef from Group OREG showed significantly higher values of a* and chroma on the 1st day of storage, coupled with lower values of hue angle, compared to Group CON (*p* < 0.05). The color parameters in Group CON remained unchanged over time, with the exception of the L* value and hue angle, which exhibited a gradual increase (*p* < 0.05). Conversely, in Group OREG, all color values increased throughout the observation period (*p* < 0.05). Consequently, on the 15th day of storage, beef from Group OREG exhibited significantly higher values of a*, b*, and chroma, along with lower values of hue angle, in comparison to Group CON (*p* < 0.05). The results of color evaluation of beef are exhibited in Table 4.

No significant differences were observed in the total amount of SFAs, MUFAs, and PUFAs between Group OREG and Group CON. However, regarding the amino acid profile, meat from Group OREG exhibited significantly higher amounts of alanine, arginine, aspartic acid, histidine, isoleucine, leucine, lysine, valine, and total amino acids, compared to meat from Group CON (*p* < 0.05). The results of fatty acids and the amino acid profile of beef are reported in Table 5.

### 3.3. Organoleptic Evaluation

**Figure 2:**
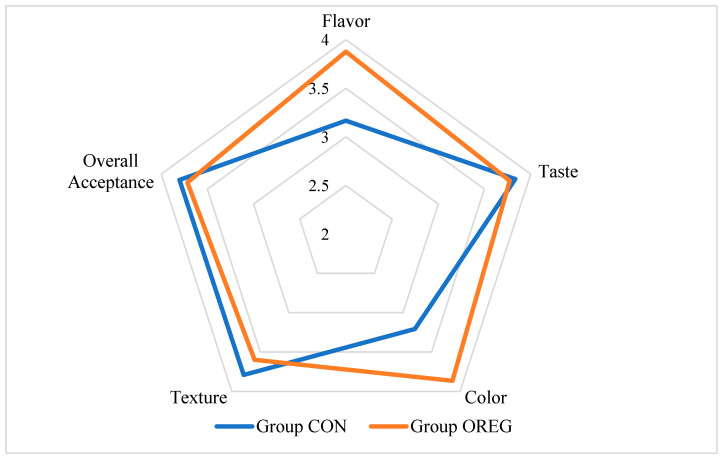
Illustration of sensory evaluation of beef from the test group (OREG) and the control group (CON).

Beef from Group OREG received a significantly higher score for flavor and color, compared to Group CON (*p* < 0.05). More specifically, the average score of flavor was 3.88 and 3.17, taste was 3.77 and 3.83, color was 3.87 and 3.21, texture was 3.60 and 3.79, and overall acceptance was 3.72 and 3.80, for Group OREG and Group CON, respectively. The results of the sensory evaluation are presented in Table 6 and Figure 2.

## 4. Discussion

Regarding the microbial counts, no significant differences were observed between carcasses from Group OREG and Group CON. This is in contrast to oregano oil’s known antimicrobial properties due to its high concentrations of carvacrol and thymol [34]. Several studies demonstrated the potential of oregano essential oil to control and inhibit the growth of microorganisms in beef [35,36]. However, other studies reported that the application of oregano oil in beef was ineffective in reducing the microbial load, whereas in some cases, it even stimulated the microbial growth [37,38]. This can be attributed to sub-lethal concentrations of essential oils or to the resistance of some microorganisms to phenolic compounds, such as thymol [39]. In our study, oregano oil was incorporated into the bulls’ diet and not directly applied to meat. Thus, oregano oil-enriched diets did not impact the microbial populations of the carcasses, perhaps due to low antimicrobial concentrations of oregano oil’s bioactive substances in meat. Hygienic conditions during slaughter continue to play an important role in determining the microbiological status of beef carcasses [40]. The population of TMVC exceeded the limits set by the European Regulation (EC) 2073/2005 on microbiological criteria for food (3.5 log_10_ CFU/cm^2^), and in some carcasses, TMVCs even surpassed the upper limit of 5.0 log_10_ CFU/cm^2^ [29]. However, the total coliform population was lower than the limits specified by the European Regulation (EC) 2073/2005 (1.5 log_10_ CFU/cm^2^). The results are in accordance with the results of Paszkiewicz and Pyz-Łukasik [41], who reported that the total aerobic bacteria count on calf carcasses slaughtered in Polish abattoirs ranged from 3.5 up to 3.85 log_10_ CFU/cm^2^. However, the coliform counts reported (1.2 log_10_ CFU/cm^2^) are larger than those in our study, possibly due to better evisceration techniques observed in the current study. In contrast, Petruzzelli et al. [42] and Camargo et al. [40] reported quite lower TMVC counts (1.96 log_10_ CFU/cm^2^ and 2.93 log_10_ CFU/cm^2^) in bovine carcasses from three small-scale Italian abattoirs and four Brazilian slaughterhouses, respectively, indicating better hygienic slaughter processes.

The pH value of meat serves as an important indicator of its overall quality, influencing factors such as color, tenderness, water retention, and taste. The reduction in meat pH is a result of lactic acid production during post-mortem glycogenolysis, originating from muscle glycogen [43]. In this study, carcasses had pH values consistently below 5.8, suggesting effective pre-slaughter handling practices [44]. No significant differences were found in the pH values between beef from Group OREG and Group CON. This aligns with findings from various studies, which indicate no pH differences in crossbred young bulls finished in feedlots, regardless of dietary supplementation with essential oils such as oregano oil, rosemary, thyme, clove, eugenol, vanillin, propolis, and others [45,46,47]. However, contrasting results were noted by He et al. [43], who reported a significant decrease in pH values of beef after dietary oregano oil supplementation, particularly at 30 min and 24 h post-slaughter. Similarly, Ornaghi et al. [48] reported that diets enriched with essential oils, including clove, castor, cashew essential oil, and combinations of thymol, eugenol, and vanillin, may decrease the pH value of beef, improve proteolysis, and enhance its tenderness. The differences observed may be attributed to variations in basal diets, essential oil dosages, environmental conditions, management practices, and the different breeds that were used in each study.

The chemical composition of beef was also evaluated. The variations between the mean measurements were relatively small, indicating uniform conditions and repeatable slaughter processes. Numerous factors, including breed, sex, nutrition, age, and the specific anatomical location of meat, can influence the chemical composition of beef [49,50]. Notably, no significant differences were found between Group OREG and Group CON. This aligns with the findings of Rivaroli et al. [44,45] and Fugita et al. [51], who reported comparable chemical compositions in beef from crossbred bulls fed essential oil blends (oregano oil, castor bean, cashew, garlic, lemon, rosemary, thyme, eucalyptus, or sweet orange) and those fed solely on the basal diet. The consistency in chemical composition is likely due to the uniformity of the basal diet across treatments, ensuring equivalent energy and protein availability for all animals.

Lipid oxidation affects meat quality and is linked to meat deterioration [52]. MDA, the primary end-product of lipid oxidation, negatively correlates with the antioxidant properties of meat [43]. In this study, meat from Group OREG exhibited significantly lower MDA levels than meat from Group CON on the 15th day of storage. This is in accordance with the results of He et al. [43], who reported that dietary supplementation of oregano oil reduced MDA concentration and increased superoxide dismutase (SOD), catalase (CAT), glutathione peroxidase (GSH-Px), and the total antioxidant capacity (T-AOC) of beef. Similarly, Ornaghi et al. [48] stated that essential oil-enriched diets decreased MDA levels in beef after seven and fourteen days of storage. Carvacrol and thymol, the active components of oregano essential oil, exhibit antioxidant properties that enhance enzyme activity against lipid oxidation, especially by scavenging free radicals, delivering electrons or hydrogen atoms, and chelating metal ions [13]. Nevertheless, Rivaroli et al. [45] noted that the addition of a high-dose essential oil blend (oregano, garlic, lemon, rosemary, thyme, eucalyptus, and sweet orange) had a pro-oxidant impact during aging, leading to higher MDA values after 14 days of storage. High doses of essential oils can permeabilize mitochondria and disrupt the mitochondrial membrane, leading to the alteration of electron flow and the production of more free radicals and reactive oxygen species (ROS). Then, ROS can oxidize lipids, proteins, and the compounds responsible for the antioxidant activity of essential oils, such as phenolic compounds. When these compounds interact with ROS, they turn into prooxidants, contributing to lipid and protein oxidation [45,53]. In this study, the MDA levels in beef increased significantly over the storage period, consistent with the findings of other studies [45,47]. The rise in MDA values is associated with meat maturation [51]. However, the observed lipid oxidation remains low in this study for all storage times, as the MDA content in beef is below 2 mg/kg, which is considered a threshold for the acceptability of oxidized meat [54].

Meat color is one of the most important factors of meat quality that affect consumers’ preferences and purchasing decisions [1]. Variations in meat color are closely tied to the amount and type of myoglobin. Oxymyoglobin is responsible for the appealing red color of fresh meat, whereas metmyoglobin, a product of oxymyoglobin and deoxymyoglobin oxidation, causes a brownish meat coloration [55]. Beef from Group OREG exhibited notably higher values for a* and chroma, along with lower values for hue angle, 24 h after slaughter. These findings indicate improved color characteristics compared to Group CON. Rivaroli et al. [45] reported that incorporating essential oils into the diet of feedlot-finished heifers had a significant impact only on the a* value of meat. Similarly, de Oliveira Monteschio et al. [47] noted that the inclusion of essential oils such as rosemary, clove, eugenol, thymol, and vanillin in the diet of young bulls increased the a* value and maintained greater redness. Higher a* and chroma values are linked to low mitochondrial respiratory activity in meat, leading to increased oxygen levels at the cut surface and the production of a vivid red color due to oxymyoglobin [47]. However, contrary observations were made by Ornaghi et al. [48] and He et al. [43], who suggested that essential oil supplementation in beef cattle diets had no significant impact on meat color. These differences may stem from variations in experimental methodologies, including different breeds, basal diets, and essential oil dosages. Furthermore, all color values of beef from Group OREG exhibited a significant increase during the 15-day storage period, unlike beef from Group CON. More specifically, on the 15th day of storage, beef from Group OREG showed significantly higher values of a*, b*, and chroma, coupled with lower values of hue angle. This implies that beef from Group OREG maintains a lighter and redder appearance on the 15th day of storage compared to Group CON. Muscle proteolysis observed during the aging process is responsible for the increase in the L* value, as the chemical structure of proteins is altered [44]. Beef is considered pale when the L* value exceeds 38.5 [44]. Given that the L* value of beef in this study exceeded 40 after two weeks of storage, the meat color of the produced beef is likely more appealing to consumers who prefer lighter meat. Additionally, oregano oil may exhibit a protective effect on myoglobin as an antioxidant. Free radicals and reactive oxygen species generated during lipid oxidation can damage the mitochondrial membrane, which negatively affects the mitochondria’s ability to reduce metmyoglobin [55]. Nevertheless, the incorporation of oregano oil may delay lipid oxidation and mitigate color deterioration due to its antioxidant activity.

The fatty acid profile influences beef flavor and nutritional value, playing a significant role in maintaining human health [49]. Fatty acids from the rumen play a crucial role in the absorption and deposition of fat in muscles [45]. Some studies suggest that dietary bioactive compounds, such as polyphenols, may modulate rumen microbiota, influence rumen biohydrogenation, and alter the fatty acid composition of meat [56]. However, no significant differences were observed in the total amount of SFAs, MUFAs, and PUFAs between beef from Group OREG and Group CON in this study, probably due to the consistent fatty acid profile of the basal diets in both Groups. Similar results were also reported by Rivaroli et al. [44,45], indicating that the addition of essential oils to the diet of crossbred bulls had minimal effects on fatty acid composition, except for myristoleic acid. However, He et al. [43] suggested that dietary oregano oil increased the concentrations of PUFAs and conjugated linoleic acid while decreasing SFA concentrations in beef. PUFAs, such as linoleic acid and linolenic acid, are essential for human health, regulating lipid metabolism, reducing cholesterol and triglyceride levels, enhancing immunity, and preventing cardiovascular diseases, atherosclerosis, and diabetes [57]. Discrepancies among studies could be attributed to the use of different breeds, diets, essential oil dosages, and management practices.

The amino acid profile significantly influences the nutritional value, flavor, and taste of beef. In this study, meat from Group OREG exhibited significantly higher levels of alanine, arginine, aspartic acid, histidine, isoleucine, leucine, lysine, valine, and total amino acids, compared to meat from Group CON. Essential amino acids, such as histidine, isoleucine, leucine, lysine, and valine, are amino acids whose carbon skeletons cannot be synthesized or are inadequately synthesized de novo by the body [58]. They have nutritional benefits for supporting growth, metabolism, and human health and they must be provided through food to meet optimal requirements [59]. Additionally, taste and flavor-related amino acids like alanine, aspartic acid, and glutamic acid contribute to the overall flavor and taste of meat, enhancing elements such as sweetness, bitterness, sourness and umami [60]. The results of this study are in accordance with the results of He et al. [43], who reported that the dietary supplementation of oregano oil significantly increased the concentrations of total amino acids, essential amino acids (including lysine, threonine, valine, methionine, and isoleucine), and flavor-related amino acids (such as glutamic acid, glycine, and alanine) in beef. This can be attributed to the fact that oregano oil reduced protein degradation in the rumen, allowing these amino acids to transfer to the small intestine for absorption [43,61]. Therefore, the incorporation of dietary oregano oil emerges as a promising approach to enhance amino acid deposition in meat, thereby improving its nutritional profile, flavor, and taste.

In the organoleptic assessment of meat, beef from Group OREG received significantly higher scores for flavor and color than Group CON. These elevated ratings can be attributed to the enhanced color parameters, improved amino acid profiles, and the heightened antioxidant capacity from the dietary incorporation of oregano oil [43,45]. In the study conducted by Wang et al. [62], the inclusion of an essential oil mixture in the diet of finishing cattle resulted in a significant improvement in juiciness and tenderness scores for beef. However, while the flavor score exhibited a numerical increase in comparison to the control group, the difference was not statistically significant. Wang et al. [62] proposed that these variations may be attributed to alterations in the muscle lipid content. Nonetheless, there is still a lack of research on how the dietary inclusion of essential oils affects meat quality and especially its organoleptic characteristics [45].

## 5. Conclusions

The results of the microbiological, physicochemical analysis, and the sensory evaluation suggest that the administration of oregano oil-enriched diets (50 mg/kg dry matter of oregano oil) in Holstein bulls improves beef quality attributes. Notably, these diets appear effective in improving color parameters and decreasing the malondialdehyde production rate and content in beef throughout the 15-day storage period, potentially extending beef’s shelf-life. Additionally, the beef displayed enhanced nutritional value and higher scores for flavor and color, confirming that consumers could recognize and prefer the improved color qualities of beef. Consequently, the incorporation of oregano oil-enriched diets is a promising method for maximizing the utility of dairy cattle and elevating the quality characteristics of the meat they produce. Further studies with a larger sample of animals and a wider range of oregano oil concentrations are needed to validate this trend, as these factors represent the main limitations of the current study. Further research is also essential to examine the impact of oregano oil-enriched diets on the performance parameters of Holstein bulls and to explore the mechanisms through which these diets improve beef quality traits.

## Figures and Tables

**Table 1:** The feed ingredients and chemical composition of the diet fed to the Holstein bulls.

**Ingredient Composition, %**	**Control**
Wheat straw	18.74
Corn grains	26.39
Barley grains	21.11
Wheat bran	13.57
Soybean meal 47	11.32
Vegetable fat	1.19
Sodium bicarbonate	0.37
Sodium chloride	0.47
Vitamins and mineral premix ^1^	1.88
Water	4.96
**Nutrient composition ^2^**	
Dry Matter (DM)	84.30
Metabolizable energy (Mcal/kg)	2.18
Crude protein, %	12.00
Crude fiber, %	12.90
Neutral detergent fiber, %	29.40
Acid detergent fiber, %	12.98
Ether extract, %	4.10
Starch, %	40.90
Sugars, %	3.80
Ca, %	0.60
P, %	0.30

^1^ Containing the following (per kg): 600,000 IU of vitamin A, 60,000 IU of vitamin D3, 2000 mg of vitamin E, 200 mg of vitamin B1, 150 mg of vitamin B2, 100 mg of vitamin B6, 40 mg of biotin, 300 mg of Fe, 300 mg of Cu, 3000 mg of Mn, 3000 mg of Zn, 15 mg of Se, 100 mg of I, and 30 mg of Co. ^2^ The chemical composition of the ingredients was calculated using the National Institute for Agricultural Research (INRA) tabulated matrix values. To validate the precision of these values, Near-Infrared Reflectance (NIR) analysis was conducted.

**Table 2:** Chemical composition (%, mean and standard deviation) of beef from the test group (OREG) and the control group (CON).

	Group CON	Group OREG	*p*
Moisture	73.77 ± 1.38	73.87 ± 1.47	0.947
Total fat	2.57 ± 0.69	2.65 ± 0.59	0.510
Total proteins	22.05 ± 0.96	22.13 ± 1.06	0.600
Collagen	1.18 ± 0.23	1.16 ± 0.24	0.849
Salt	0.92 ± 0.25	0.99 ± 0.43	0.709
Ash	0.77 ± 0.29	0.80 ± 0.32	0.698

**Table 3:** The pH and malondialdehyde content (ng/g, mean and standard deviation) of beef from the test group (OREG) and the control group (CON) (*p* < 0.05 in bold).

	Group CON	Group OREG	*p*
pH (day 1)	5.56 ± 0.15	5.61 ± 0.15	0.261
pH (day 15)	5.58 ± 0.16	5.62 ± 0.15	0.432
*p*	0.383	0.505	
MDA * (day 1)	47.92 ± 20.30	33.31 ± 14.97	0.055
MDA (day 15)	105.91 ± 39.40	68.52 ± 34.40	**0.010**
*p*	**<0.001**	**0.002**	

* MDA: malondialdehyde.

**Table 4:** Color values (mean and standard deviation) of beef in the test group (OREG) and the control group (CON) (*p* < 0.05 in bold).

		Group CON	Group OREG	*p*
L*	day 1	38.23 ± 1.90	38.46 ± 2.24	0.698
	day 15	41.43 ± 2.21	41.98 ± 2.52	0.426
	*p*	**<0.001**	**<0.001**	
a*	day 1	18.09 ± 1.25	20.84 ± 1.22	**<0.001**
	day 15	17.95 ± 0.82	23.21 ± 1.78	**<0.001**
	*p*	0.612	**<0.001**	
b*	day 1	6.44 ± 1.37	5.87 ± 0.91	0.107
	day 15	7.01 ± 1.17	8.06 ± 1.21	**0.003**
	*p*	0.121	**<0.001**	
Chroma	day 1	19.22 ± 1.62	21.70 ± 1.20	**<0.001**
	day 15	19.29 ± 1.12	24.58 ± 2.03	**<0.001**
	*p*	0.839	**<0.001**	
Hue angle	day 1	19.39 ± 2.62	15.78 ± 2.34	**<0.001**
	day 15	21.22 ± 2.65	19.07 ± 1.68	**0.001**
	*p*	**0.021**	**<0.001**	

**Table 5:** Fatty acids (%, mean and standard deviation) and amino acid profile (g/kg, mean and standard deviation) of beef from the test group (OREG) and the control group (CON) (*p* < 0.05 in bold).

	Group CON	Group OREG	*p*
**Fatty acids**			
SFAs *	51.10 ± 6.71	54.52 ± 5.62	0.513
MUFAs *	45.83 ± 6.85	41.98 ± 5.39	0.275
PUFAs *	3.04 ± 0.31	3.50 ± 0.36	0.127
**Amino acids**			
Alanine	7.72 ± 0.55	9.46 ± 0.22	**0.007**
Arginine	12.81 ± 0.29	16.86 ± 1.32	**0.007**
Aspartic acid	12.62 ± 0.65	16.12 ± 1.75	**0.031**
Glutamic acid	9.74 ± 2.08	12.83 ± 0.23	0.121
Glycine	11.28 ± 0.93	13.68 ± 1.50	0.077
Histidine	6.59 ± 0.48	8.81 ± 0.94	**0.022**
Isoleucine	6.22 ± 0.29	7.91 ± 0.74	**0.045**
Leucine	12.54 ± 0.65	14.42 ± 0.49	**0.016**
Lysine	11.37 ± 0.22	12.72 ± 0.54	**0.015**
Methionine	3.83 ± 0.45	4.48 ± 0.16	0.080
Phenylalanine	8.23 ± 0.88	6.92 ± 0.13	0.064
Proline	11.88 ± 1.35	12.88 ± 2.30	0.553
Serine	7.21 ± 0.60	7.84 ± 1.47	0.530
Threonine	5.66 ± 0.11	6.00 ± 0.89	0.552
Tyrosine	4.91 ± 0.58	4.84 ± 0.36	0.854
Valine	7.72 ± 0.44	9.95 ± 0.28	**0.002**
Total amino acids	140.30 ± 1.53	165.00 ± 3.00	**<0.001**

* SFAs: total amount of saturated fatty acids; MUFAs: total amount of monounsaturated fatty acids; PUFAs: total amount of polyunsaturated fatty acids.

**Table 6:** Organoleptic evaluation (mean and standard deviation) of beef from the test group (OREG) and the control group (CON). (*p* < 0.05 in bold).

	Group CON	Group OREG	*p*
Flavor	3.17 ± 0.36	3.88 ± 0.44	**<0.001**
Taste	3.83 ± 0.18	3.77 ± 0.56	0.728
Color	3.21 ± 0.31	3.87 ± 0.47	**0.001**
Texture	3.79 ± 0.25	3.60 ± 0.62	0.736
Overall Acceptance	3.80 ± 0.23	3.72 ± 0.48	0.861

## Data Availability

Data presented in this study are contained within the article.
